# Paper waste and carbon emissions from oral contraceptive leaflets

**DOI:** 10.1371/journal.pone.0312505

**Published:** 2024-10-30

**Authors:** Myriam Safrai, Dana Elly, Noah Gruber, Benjamin Stern, Doron Kabiri, Moran Shapira

**Affiliations:** 1 IVF unit, Department of Obstetrics and Gynecology, Chaim Sheba Medical Center, Faculty of Health Science and Medicine, Tel Aviv University, Tel Aviv, Israel; 2 Faculty of Medicine, Technion-Israel Institute of Technology, Haifa, Israel; 3 Pediatric Endocrine and Diabetes Unit, Edmond and Lily Safra Children’s Hospital, Chaim Sheba Medical Center, Ramat-Gan, Israel, Faculty of Medicine, Tel Aviv University, Tel Aviv, Israel; 4 Anterior Segment and Refractive Surgery Department, Rothschild Foundation Hospital, Paris, France; 5 Department of Ophthalmology, Hadassah Hebrew Medical Center and Faculty of Medicine, Hebrew University of Jerusalem, Jerusalem, Israel; 6 Department of Obstetrics and Gynecology, Hadassah Hebrew Medical Center and Faculty of Medicine, Hebrew University of Jerusalem, Jerusalem, Israel; VIT University, INDIA

## Abstract

Oral contraceptives (OC) are the most used form of contraception among women in the U.S. and Europe. Like other medications, their packaging must include patient information leaflets. This study quantifies the environmental impact of paper waste generated by these leaflets. We conducted an observational analysis, measuring the weight of leaflets, pills, and packaging components across various OC brands. Significant variations in leaflet weights were observed. On average, leaflets accounted for 55% of the package weight, while pills and blister dispensers represented only 32%. The mean weight of OC leaflets was 12.3 ± 5.5 grams (4.7–21.9 grams), leading to an estimated annual paper waste of 6,118.4 tons, 5,763.5 tons of carbon dioxide equivalent emissions, and the use of approximately 146,841 trees for production. Standardizing leaflet weight to the lightest reported can reduce annual waste by 3780.5 tons of paper. This study highlights the substantial environmental cost of the waste generated from OC leaflets and proposes practical strategies to mitigate waste, including electronic leaflets and standardized packaging. Targeting these materials presents a significant opportunity to enhance sustainability, aligning with global efforts to reduce greenhouse gas emissions from the healthcare sector.

## Introduction

Oral contraceptives (OC) are used by 17.8% of reproductive aged women, making them the most popular form of contraceptive in the U.S. and Europe [1]. To ensure that patients are fully informed about their medication, the Food and Drug Administration (FDA) mandates the inclusion of both IFU (instructions for use) and PPI (patient package insert) in each OC prescription in the U.S., while the European Medicines Agency (EMA) has similar requirements for Europe [2–4]. The IFU outlines essential steps for a drug’s preparation, administration, and disposal, while the PPI provides patient-specific details. Together, they form the drug leaflet, which is printed on paper and consists of multiple pages translated into various languages. This information is proposed by the manufacturer, reviewed, and approved by the regulator to ensure it is standardized and accessible. However, printed information provided with medications does not necessarily enhance patient knowledge, as many individuals do not value the written information they receive with their medicine and forgo reading it [5] This may be especially true for long-term users, who have been shown to comprise over half of OC users [6].

While indispensable for human well-being, the medical sector is one of the highest contributors to carbon emissions in developed countries [7, 8]. It accounts for approximately 4.5% of global greenhouse gas (GHG) emissions and a similar percentage of harmful air pollutants [7, 9, 10]. This waste generation results from using single-use equipment, unnecessary procedures, and challenges that vary by geographic location and medical specialty [11–13]. Specifically, the pharmaceutical industry is estimated to be responsible for more than 50% of the U.S. national healthcare GHG emissions [14]. Drug-related waste arises from several factors, such as leftover medication packing forms, drug multiple-dose formats, and included IFU contributing to paper waste [15–18]. Specifically, the lifecycle of paper waste contributes to GHG emissions through energy-intensive production processes (e.g. tree harvesting, pulping, and papermaking), transportation, and methane generation from landfill disposal. A recent study has shown that IFUs in intraocular lens packages for cataract surgery generate about 128 tons of paper waste yearly, equal to 120 tons of carbon dioxide equivalent (CO_2_eq) emissions [15]. Streamlining IFU brochures may reduce its contribution to GHG emissions, leading to sustainable adaptations towards climate change. Financial factors can also strongly motivate a change in IFUs provision. The pharmaceutical packaging and labeling market in Europe is projected to reach $35.78 billion by 2028, with paper accounting for the largest segment at 40% in 2022 [19]. Reducing printed leaflets can thus lead to direct savings in packaging and printing costs.

Despite considerable research efforts in the health industry and organizational pledges to decrease emissions and improve the environmental sustainability of healthcare, there are no previous publications addressing packaging optimization of commonly prescribed drugs such as OCs. In this study, we aimed to quantify the amount of paper used for OC leaflets and its CO_2_eq. By quantifying this amount of paper, our research will provide a tangible measure of the environmental impact of these pharmaceutical products. This study highlights the environmental burden of potentially unnecessary packaging and underscores the potential benefits of alternative packaging solutions, which can contribute to reduced carbon emissions and improved environmental sustainability.

## Methods

### Study design

This observational analytical study included second to fourth-generation progestin-only and combined oral contraceptives, with the latter involving both monophasic and multiphasic preparations. As of January 2024, a total of 22 such OCs were marketed in Israel. Five were unavailable at pharmacies at the time of the study’s analysis. Thus, a total of 17 OCs were included in the study. For each OC, 3 separate packages, along with their corresponding components, were sampled. Each component was weighed three times. The final figure presented for each component in Table 1, was the average of all 9 measurements. An assessment was performed by the authors to verify the inclusion of pills originating from different manufacturers and components.

**Table 1:** Weight components of oral contraceptive packaging.

Supplier, Brand	Components	Languages (number)	QR inclusion, location	Weight (grams)					Pills/ leaflet	Pills/(box + leaflet) (%)
				Leaflet	Box	Additional package	Blisters package and pills	Total package		
Bayer, Melian	Gestodene, Ethinylestradiol	3	Yes, IFU	13.05	5.16		3.05	21.26	0.23	16.77
Bayer, Diane 35	Cyproterone, Ethinylestradiol	3	Yes, IFU	15.35	5.12		3.17	23.64	0.21	15.50
Bayer, Yaz	Drospirenone, Ethinylestradiol	3	Yes, pill blisters, IFU	14.14	5.19	0.40**	6.42	26.14	0.46	33.24
Bayer, Qlair	Dienogest	3	Yes, pill blisters, IFU	14.38	5.10	0.43**	6.40	26.31	0.44	32.85
Bayer, Yasmin	Drospirenone, Ethinylestradiol	3	Yes, box	18.19	5.11	1.96***	2.91	28.17	0.16	12.51
Bayer, Gynera	Gestodene	3	Yes, box	18.34	5.18		3.11	26.64	0.17	13.24
Organon, Mercilon	Desogestrel, Ethinylestradiol	3	No	21.85	5.06	3.28*	3.27	33.46	0.15	12.14
Pfizer, Harmonet	Gestodene, Ethinylestradiol	3	No	11.54	5.36		3.35	20.25	0.29	19.85
Theramex, Zoley	Nomegestrol, Estradiol	3	No	20.20	8.82	3.79***	4.08	36.88	0.20	14.05
Pfizer, Minesse	Gestodene, Ethinylestradiol	3	No	11.59	7.58		4.28	23.45	0.36	22.33
Dexcel Pharma, Fominic	Desogestrel	3	No	4.73	4.90		4.16	13.80	0.88	43.22
Organon, Cerazette	Desogestrel	3	No	10.77	5.48	1.21*	3.36	20.82	0.32	20.70
Bayer, Visabelle	Dienogest	3	No	4.77	5.14		3.49	13.40	0.80	35.22
Pfizer, Minulet	Gestodene, Ethinylestradiol	3	No	11.74	5.48		3.36	20.58	0.29	19.53
Gedeon Richter, Belara	Chloramadinone, Ethinylestradiol	3	No	4.74	5.07		3.74	13.55	0.80	38.14
Teva, Diamilla	Desogestrel	3	No	5.72	5.32		4.19	15.23	0.71	37.90
Dexel Pharma, Feminet	Desogestrel, Ethinylestradiol	3	No	7.32	4.51		3.64	15.47	0.50	30.81

*aluminum

**plastic

***holder

IFU–Information for user

The following packages contained 2 or 3 pill blister packages: Fominic, Minulet, Diamilla

### Data sources and measurement

The study involved detailed weight measurements of leaflets, pills with their direct blister dispensers, packaging, and additional products provided with each OC package by the manufacturer. Measurements were conducted using a high-precision balance (AUY120 analytical Shimadzu scale). Recalibration was performed before each measurement to ensure accuracy. This study was exempted from the institutional review board review as it was observational and did not involve collecting or analyzing identifiable patient data.

### Statistical methods

Quantitative analysis was conducted to assess the weight of the leaflets and associated materials. The recorded weights of the leaflets and other package components were then entered into Microsoft Excel version 2403 and computed using GraphPad Prism version 10. We computed each variable’s mean weight, standard deviation, and range. Additional calculations were made to assess the relation between different package components. This GraphPad Prism software was also employed to generate graphical representations of various weight components.

### Paper waste, CO_2_eq emissions, and cost assessment

Using the calculated mean weight of the leaflets and the estimated number of OC users in the U.S. and Europe, we estimated the production of leaflets and the annual paper waste generated from printing OC leaflets in both regions. This assessment involved calculating the average mass of these leaflets alongside the evaluation of the monthly prevalences of OC users.

### Estimation of OC utilization rate

We relied on comprehensive data delineating the utilization rates of contraceptive methods by women aged 15–49 years worldwide by region [1]. This resource provided estimates on the utilization rate of different contraceptive methods in reproductive aged women, drawn from a comprehensive dataset of 1,247 surveys covering 195 countries and regions [20]. It was discerned that approximately 152 million women globally used OCs in 2019, with about 41.5 million users in the U.S. and Europe [1]. Notably, another database reported a similar global figure of 151 million OC users, reinforcing the validity of the estimation [21]. Since most women use OCs for extended periods and these drugs are typically distributed in monthly packages, we multiplied the number of users by 12 to account for the annual number of utilized OC packages [6].

### Estimation of CO_2_eq emissions and paper waste

We evaluated the CO_2_eq emissions associated with this process, given that producing 1 ton of paper leads to 942 kg of CO_2_eq emissions [22, 23]. We also took into account the number of reams of copy paper this represented, noting that 1 ton of paper equals 400 reams [22, 24]. Furthermore, we estimated the number of trees required to produce the amount of paper used for pharmacological leaflets. Guidelines and manufacturers recommend uncoated virgin paper to enhance readability by reducing transparency and glare, making small text easier to read [25, 26]. Our study used the metric of 24 trees to produce one ton of this type of paper. [24].

### Cost estimation

The cost of manufacturing an individual leaflet is dictated by the costs of paper, ink, printing, and labor. The cost of each component may vary depending on the production location and leaflet’s size. A detailed European analysis estimated the average cost of a single leaflet to be approximately 0.95 Euros [4].

## Results

A total of 17 OCs were analyzed. The mean weight of the leaflets was 12.3±5.5 grams (ranging from 4.7 to 21.9 grams), and the average weight of the pills and blister dispensers was 3.9±1.0 grams (ranging from 2.9 to 6.4 grams). The mean weight of the boxes was 5.5±1.1 grams (ranging from 4.5–8.8 grams). The weight of the components included in the different OC packages and the relation between them are reported in Table 1.

In 2019, the number of OC users in the U.S. and Europe was estimated at 41.5 million, translating to an annual usage of approximately 497.4 million OC packages, each accompanied by an informational leaflet [1]. When calculated on a cumulative basis, the annual weight of these leaflets, distributed globally with oral contraceptive packages, totals approximately 6,118.4 tons. This production process results in the emission of 5,763.5 tons of CO_2_eq. The amount of paper used corresponds to approximately 2,447,356.4 reams of copy paper and requires about 146,841.4 trees for annual production. If extrapolated globally, with an estimated 152 million OC users worldwide, this results in an annual production of approximately 1,824 million OC packages. This production requires approximately 22,435.2 tons of paper, generates 21,134.0 tons of CO2 equivalents, and necessitates 538,444.8 trees for manufacturing.

**Fig 1:**
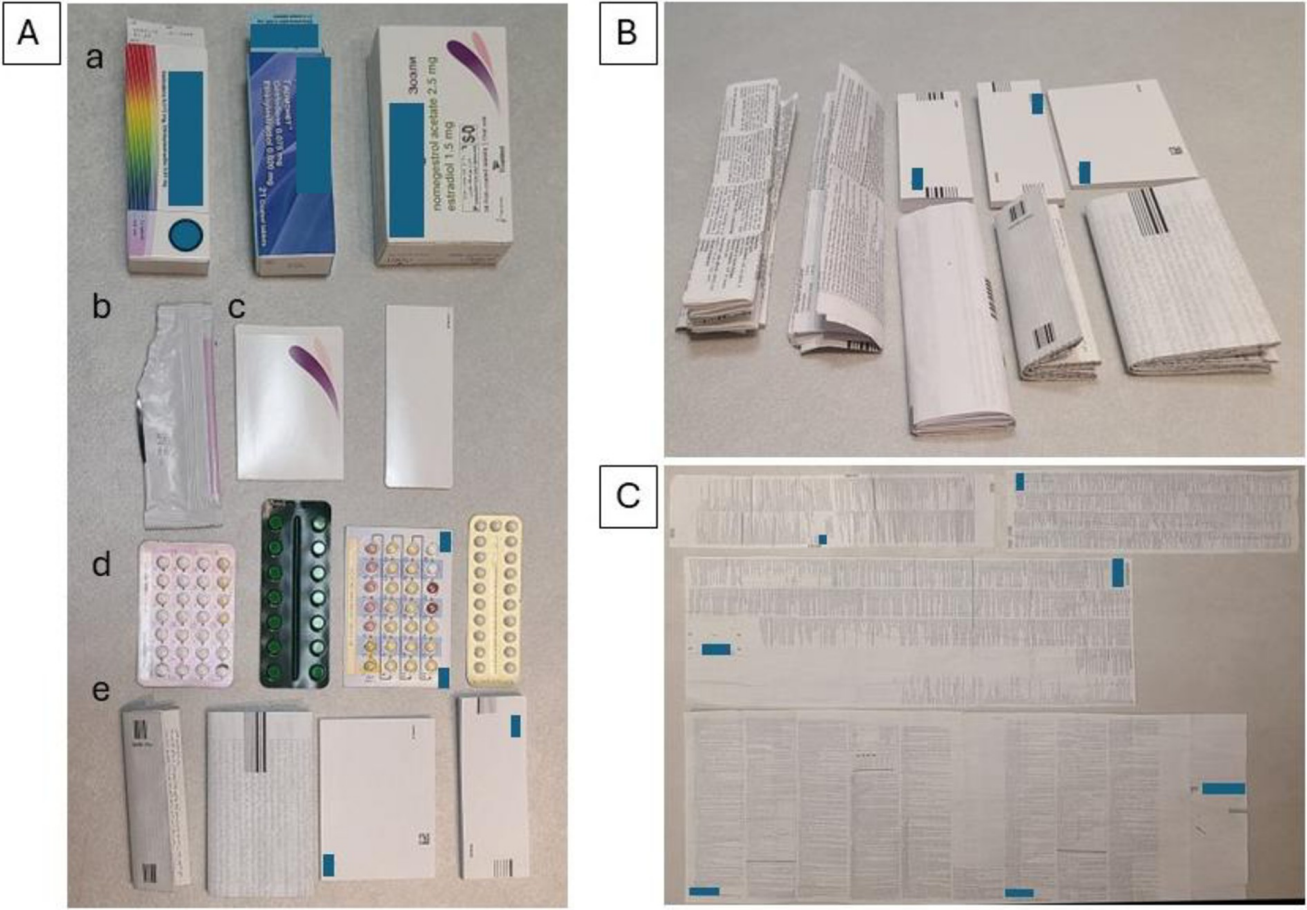
Overview of different products and packaging provided with oral contraceptives (OC). A. Various forms of products provided with OC: a. boxes, b. aluminum foil wrapping, c. Pill holder, d. blister pills dispenser, e. Leaflets B. Diversity in Leaflets Accompanying OC: I. Lightest leaflet (4.73 grams) as Observed in Our Study, II. Heaviest leaflet (21.85 grams) recorded in This Study. C. Variation of leaflets accompanying oral contraceptives (OC): The upper section displays lighter leaflets, while the lower section shows heavier leaflets, including more empty spaces.

A detailed analysis of the components within the OC packages revealed that four of the seventeen OCs contained additional packaging, either aluminum or a reusable holder (**Fig 1A**). Additionally, two OCs had a distinct packing method: the pills were folded within a cardboard package and wrapped with an additional plastic wrap.

**Fig 2:**
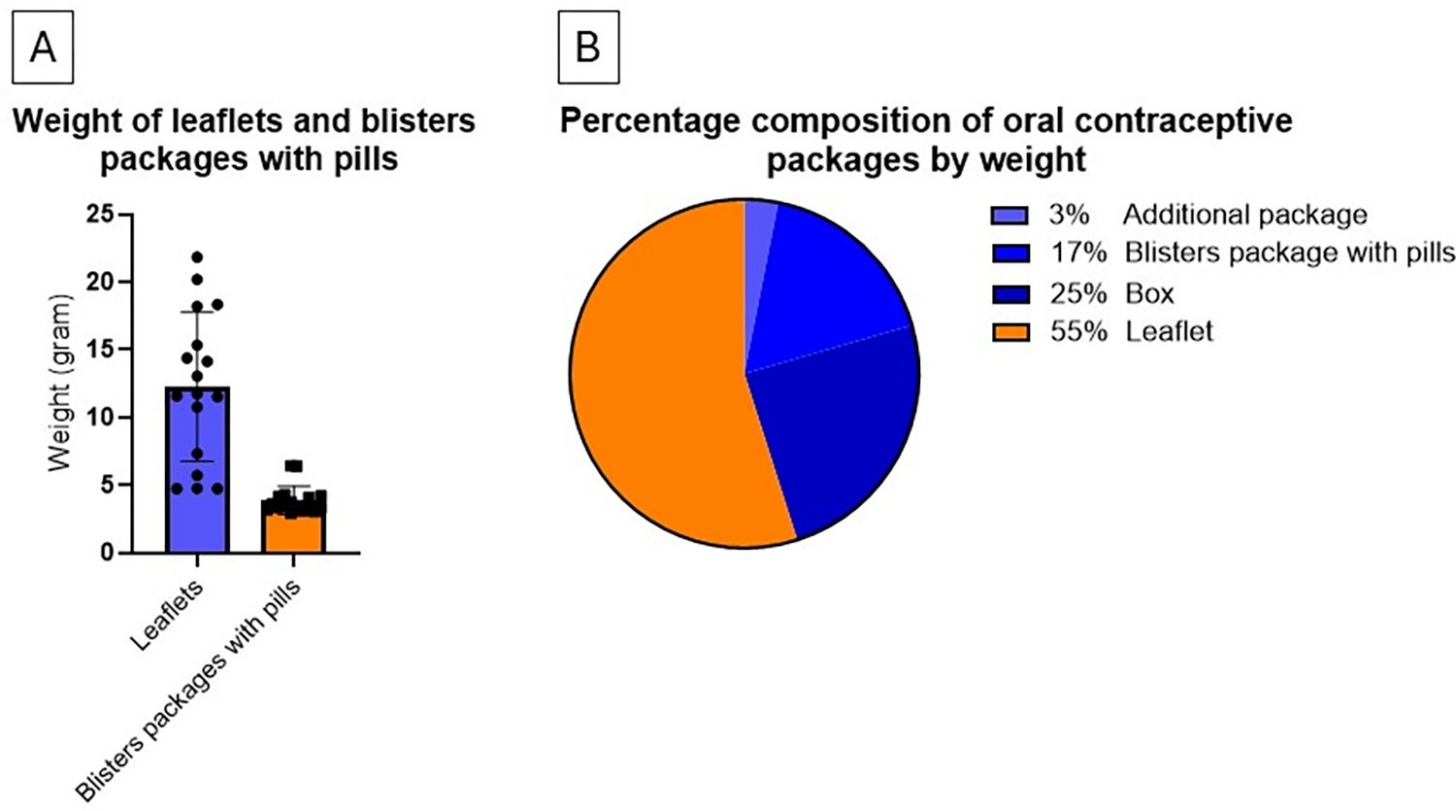
Weight distribution of components in oral contraceptive (OC) packages. A: Weight distribution of leaflets and pills in blister in OC packaging. The average weight of the leaflets exceeds that of the pills, including their blister packaging. Notably, there is significant variability in leaflet weights, whereas pill weights are more uniform and generally lighter. B: Weight distribution of various components within the OC packages. The leaflet accounts for over half of the total weight, and the box a quarter.

As shown in **Fig 1B and 1C**, there is a considerable variation in the leaflets and their weights. Conversely, our measurements reveal that the pills’ weight and packaging are consistently similar (**Fig 2A**). The heaviest leaflet was 4.6 times heavier than the lightest. Both, along with others of intermediate weight, complied with existing regulations and were approved for use. All leaflets included the same number of languages and similar font sizes. Differences in weight were primarily attributed to variations in design and content. For instance, the lightest leaflet consisted of a single folded large sheet of paper with brief text devoid of graphical elements or significant empty space.

In contrast, the heaviest leaflets featured a larger single page folded alongside longer information and sometimes included tables or graphics such as flowcharts or pill dispenser diagrams (**Fig 1C**). These heavier leaflets often had varying amounts of empty space. Another notable category of heavier leaflets was small booklet-style leaflets, which displayed a mix of tables and flowcharts. No empty pages were observed in those leaflets.

Adopting the lightest format of leaflets in the U.S. and Europe, weighing 4.7 grams instead of using a variety of leaflets with an average weight of 12.3 grams, will result in using 2,337.9 tons of paper for OC leaflets. Therefore, this change can already save 3780.5 tons of paper annually, 3561.2 tons of CO_2_eq gas emitted, and 90,731.3 trees.

Six of the seventeen OCs studied, representing 35% of the sample, featured a quick response (QR) code distributed across different components of the packages (Table 1). Notably, all these QR codes were printed on Bayer AG (Germany) products. The QR codes appeared not for public use, as they do not provide access to information upon scanning, and all relevant details were provided in the accompanying leaflets.

OC pills are dispensed in user-friendly blister packaging, designed to facilitate patient monitoring, ensure adherence, and prevent missed doses. However, our findings underscore the significantly lower weight of OC pills and their dispensary packaging, ranging between 2.9 to 6.4 grams, in stark contrast to the leaflets and other packaging elements. The aggregate weight of the medication alongside its packaging is only ~32% of the leaflet alone, and it represents 17% of the entire package’s weight, encompassing boxes, leaflets, and any extra materials provided by the manufacturer. The leaflet represents, on average, 55% of the total package weight. (**Fig 2B**).

The average cost of an individual leaflet is 0.95 Euros. An annual production of 497.4 million OC packages results in a total expense of 472.5 million Euros each year.

## Discussion

The current study is the first to evaluate the paper waste of the leaflet included in packages of OCs, one of the most popular contraceptives used worldwide. Our study shows that the mean weight of OC leaflets is 12.3±5.5 grams. While this individual leaflet weight may seem insignificant, their cumulative weight becomes substantial when considering the estimation of 41.5 million monthly users in the U.S. and Europe, leading to a significant paper waste of more than 6,110 tons per year and generating more than 5,760 tons of CO_2_eq. To put this into context, this waste is the equivalent of almost 2.45 million reams of copied paper, requiring harvesting approximately 147,000 trees. Additionally, these results entail significant environmental costs related to paper production, including waste disposal, energy consumption, and the transportation of the leaflets.

On Earth Day 2022, the White House and the Department of Health and Human Services launched the Health Sector Climate Pledge to cut emissions by 50% by 2030 and reach zero emissions by 2050 [27]. Previous studies investigated the possibility of reducing waste in the pharmacological industry in various ways. For example, a study found that redispensing unused oral anticancer drugs can reduce waste by 68.1% [28]. Similarly, studies have shown that reducing the space between blister packs in drug packaging can reduce waste by over a third [18]. Moreover, using electronic IFU in packaging intraocular lenses for cataract surgery was found to potentially decrease CO_2_eq by 84% [15]. An additional study analyzing waste from intraocular lens packaging identified IFUs as the largest component within the boxes [29]. Similarly, our study found that leaflets constitute more than half of the total weight of OC packaging. This underscores the potential for significant waste reduction by transitioning to more sustainable alternatives.

Our analysis reveals the notably low weight of OC pills and their blister packaging compared to the other package components. This emphasizes the substantial environmental benefits of packaging modifications, especially for OC in the pharmaceutical industry. Since lighter packaging can significantly reduce the ecological footprint from transportation emissions, shifting to lightweight packaging could markedly decrease paper waste and drug distribution’s overall financial and environmental costs [30].

An ultimate goal for reducing paper waste and moving forward with substantial alternatives is promoting the switch to QR codes, a strategy already mandated in Brazil [31]. This approach has also begun to be implemented in various European countries, specifically for drugs provided in hospitals. Using QR codes instead of paper leaflets may also lead to more environmentally sustainable packaging, secondary to reducing packaging size [30]. For instance, switching to cardboard holders included in some standard packaging and printing QR codes on these compact packages can significantly reduce waste. This innovation not only moves toward substantial waste reduction but also opens avenues for enhanced patient education. QR codes enable easy updates of drug information and allow the use of multimedia resources such as videos, which have been shown to improve patients’ understanding and care quality [32–34]. It is pertinent to recognize that OCs are predominantly utilized by a younger demographic, typically aged 15–45. This group is generally more adept with electronic media and is already using the Internet to search for medical information, suggesting that OCs are a prime candidate for implementing electronic leaflets (e-leaflets) [35].

Nevertheless, transitioning to e-leaflets presents several challenges. Firstly, regulatory bodies such as the FDA and EMA oversee leaflet regulations. Pharmaceutical companies should obtain regulatory approval for e-leaflets and ensure that QR codes meet accessibility and security standards. They should also demonstrate that the technology can maintain and update these digital formats. Collaborating with regulatory agencies can help standardize packaging globally, potentially reducing environmental impact and enhancing patient access to updated, interactive medical information. Secondly, the implementation of e-leaflets marks a significant shift that should be supported by consumer education. Efforts should focus on raising awareness of digital formats, teaching users how to access e-leaflets via QR codes, and providing clear instructions for all levels of technological literacy. Lastly, it should be noted that some regions have limited internet access, complicating the adoption of e-leaflets. This necessitates exploring alternatives for these demographics and developing transition strategies.

The current study demonstrates a large variation in the weight of leaflets compared to the uniform weight of pills and dispensers. Our analysis indicates that standardizing all leaflets in the U.S and Europe to the weight of the lightest one currently in use could potentially decrease paper waste by more than 3,700 tons annually, reducing by more than 3,500 tons the production of CO_2_eq and sparing almost 91,000 trees. This finding aligns with previous research, showing that reducing material and volume in pharmaceutical packaging significantly lessens its environmental impacts [36].

CO_2_eq and other greenhouse gas emissions are key drivers of climate change and rising global temperatures, posing significant threats to human life [37]. For instance, there is a clear correlation between increasing CO2 concentrations and global temperature rise over the years [38]. As such, redesigning leaflets to be lighter may be a practical approach for countries where the infrastructure may not yet support fully digital solutions. In developed countries, it may also serve as an interim solution until E-leaflets become a standard. This approach is similar to practices adopted in Japan, where the digitalization of package inserts has been implemented for products used by healthcare professionals in medical institutions and pharmacies. During the transition period, paper leaflets were still included alongside QR codes to facilitate the transition to digital formats [39]. In addition, such a change will align with the previously advocated standardization and simplification of OC labels, thereby simplifying switching between different OCs [40].

E-leaflets offer clear environmental benefits, but their advantages extend beyond sustainability. Globally, they facilitate the use of a standardized packaging format that can link to diverse and tailored information in multiple languages, potentially increasing the accessibility of medicines across international borders [30]. This uniform approach enables the storage of a single, lighter stock of packaging, which can significantly reduce the costs associated with drug manufacturing, storage, and distribution. Manufacturing millions of OC leaflets results in more than 472 million Euros yearly. The long-term cost reduction benefits are profound, as they may help mitigate drug shortages and foster the introduction of new pharmaceuticals in the market [39, 41].

On a global scale, most countries have regulatory agencies that still require paper leaflets to be included with pharmacological drugs sold to the public and have similar requirements across different regions [2–4, 42–44]. Therefore, we theorize a significant global impact if everyone had to follow the same requirement, resulting in the same average weight for OC package inserts. As OCs are the third most common method of contraception worldwide, the number of OC users is substantial, resulting in over 1,824 million annual OC packages. This production demands approximately 22,435 tons of paper, generates 21,134 tons of CO2 equivalents, and requires almost 538,445 trees for manufacturing. While we acknowledge that this is only an extrapolation, future studies could aim to collect data from other regions to validate this approximation.

Several limitations of the current study should be acknowledged. First, our calculations are based on estimated values for the annual number of OCs used in the U.S., Europe, and worldwide based on the tree-to-paper production ratio. The exact number of OCs is unknown; however, the estimations were all based on available literature and were considered sufficiently accurate [1]. In addition, the calculation of CO_2_eq emissions was made based on the general emission from paper production, and the generalizability of such figures was not evaluated previously. Therefore, a specific study involving pharmacological leaflets, specifically OC, may provide broader information about their impact on sustainability. Second, our sample consists of major international manufacturers available in a specific region, which may limit its generalizability to the broader market. However, similarities in the types of OCs marketed worldwide and the consistent regulatory mandates for pharmacological leaflets across different countries support the broader applicability of our findings [45]. Third, our data did not account for recycling; it is difficult to determine the percentage of recycled paper. Yet, it is important to consider that pharmaceuticals are commonly discarded with domestic waste and not recycled, resulting in a genuine loss of paper and wood resources [36, 46]. Future research could attempt to investigate the actual fraction of leaflets recycled. In addition, we did not consider the variation in the use of each OC brand that may influence the leaflet’s waste or the use of combined packages, including a drug supply, for three months. It is important to note that the distribution of multipack drugs has been recognized to contribute to pharmacological waste in other ways [17].

## Conclusion

This is the first study to demonstrate the significant environmental cost and paper waste generated by information leaflets included in OC packages. Our results underscore the considerable global impact of seemingly minor components such as a single leaflet. Reducing OC leaflet weight offers a valuable opportunity to enhance sustainability and support global efforts to reduce greenhouse gas emissions in healthcare. Practical solutions, like electronic leaflets and standardized packaging, could lower the environmental footprint while maintaining regulatory compliance and patient information access. Future research could explore consumer acceptance of electronic leaflets, evaluate regulatory adjustments required for implementation, and investigate strategies to promote their broader adoption across the industry.

## Supporting information

S1 Table Details, components, and weight of oral contraceptive packages from different brands. The table presents information on suppliers, active ingredients, packaging languages, and weights of components included in oral contraceptive packages. Weights are shown across replicates.
(XLSX)
